# Identification of novel cerebellar developmental transcriptional regulators with motif activity analysis

**DOI:** 10.1186/s12864-019-6063-9

**Published:** 2019-09-18

**Authors:** Thomas J. Ha, Peter G. Y. Zhang, Remi Robert, Joanna Yeung, Douglas J. Swanson, Anthony Mathelier, Wyeth W. Wasserman, Sujin Im, Masayoshi Itoh, Hideya Kawaji, Timo Lassmann, Carsten O. Daub, Erik Arner, The FANTOM Consortium, Piero Carninci, Yoshihide Hayashizaki, Alistair R. R. Forrest, Daniel Goldowitz

**Affiliations:** 1 Centre for Molecular Medicine and Therapeutics at the BC Children’s Hospital Research Institute, Department of Medical Genetics, University of British Columbia, Vancouver, BC Canada; 2 Centre for Molecular Medicine Norway (NCMM), Nordic EMBL Partnership, University of Oslo, 0318 Oslo, Norway; 3 Department of Cancer Genetics, Institute for Cancer Research, Oslo University Hospital, Radiumhospitalet, 0372 Oslo, Norway; 4 RIKEN Omics Science Center (OSC), 1-7-22 Suehiro-cho, Tsurumi-ku, Yokohama, 230-0045 Japan; 5 RIKEN Center for Life Science Technologies, Yokohama, Japan; 6 RIKEN Preventive Medicine and Diagnosis Innovation Program, Wako, Japan; 7 Telethon Kids Institute, The University of Western Australia, 100 Roberts Road, Subiaco, Subiaco, Western Australia 6008 Australia; 8 Division of Neurology, Department of Pediatrics, University of British Columbia and BC Children’s Hospital, Vancouver, BC Canada

**Keywords:** Cerebellar development, Transcriptome, HeliScopeCAGE, Motif activity, Transfactivity, Transcription factors, RNAi, Atf4, Rfx3, Scrt2

## Abstract

**Background:**

The work of the FANTOM5 Consortium has brought forth a new level of understanding of the regulation of gene transcription and the cellular processes involved in creating diversity of cell types. In this study, we extended the analysis of the FANTOM5 Cap Analysis of Gene Expression (CAGE) transcriptome data to focus on understanding the genetic regulators involved in mouse cerebellar development.

**Results:**

We used the HeliScopeCAGE library sequencing on cerebellar samples over 8 embryonic and 4 early postnatal times. This study showcases temporal expression pattern changes during cerebellar development. Through a bioinformatics analysis that focused on transcription factors, their promoters and binding sites, we identified genes that appear as strong candidates for involvement in cerebellar development. We selected several candidate transcriptional regulators for validation experiments including qRT-PCR and shRNA transcript knockdown. We observed marked and reproducible developmental defects in Atf4, Rfx3, and Scrt2 knockdown embryos, which support the role of these genes in cerebellar development.

**Conclusions:**

The successful identification of these novel gene regulators in cerebellar development demonstrates that the FANTOM5 cerebellum time series is a high-quality transcriptome database for functional investigation of gene regulatory networks in cerebellar development.

**Electronic supplementary material:**

The online version of this article (10.1186/s12864-019-6063-9) contains supplementary material, which is available to authorized users.

## Background

Brain development requires intricately controlled expression of specific gene regulatory networks across time. Despite recent developments in genomics technology, large-scale transcriptome analyses across time in neural development is limited. The cerebellum is a less complex, anatomically discrete and well-studied part of the mammalian brain that lends itself to such an analysis. The FANTOM5 Consortium has recently provided a novel examination, *en masse*, of the genomic factors that are in play as cells and tissues differentiate or transition from one state to the next in both human and mouse time course data [1]. The HeliScopeCAGE technology, employed by the FANTOM5 Consortium, combines the CAGE (Cap Analysis of Gene Expression) protocol and Helicos sequencing to produce direct, high-precision measurement of transcription based on 5′ end sequence of capped RNA [2]. Included in this dataset was tissue from the mouse cerebellum that was examined at 24 h intervals throughout its development beginning at E12 (embryonic day 12) to P0 (postnatal day 0) and at 72 h intervals from P0 to P9. These developmental time windows span most of the important neurodevelopmental events such as cell specification, emergence from the cell cycle, differentiation, migration and maturation in major neuronal types of cerebellum including the cerebellar granule cells, Purkinje cells, cerebellar interneurons, and cerebellar nuclear neurons.

There is a limited number of neuronal types that colonize the cerebellum and they originate from two separate zones: the rhombic lip and the ventricular neuroepithelium. The rhombic lip (RL) gives rise to the excitatory neurons of the cerebellum that include the glutamatergic cerebellar nuclear neurons, the granule cell precursors, and the unipolar brush cells. The ventricular neuroepithelium gives rise to Purkinje cells and other GABAergic interneurons and cerebellar nuclear neurons [3–5]. The key transcription factors (TFs) Atoh1 and Pax6 are expressed in RL and the external germinal layer (EGL) [6–8]; and Ptf1a is expressed in the ventricular neuroepithelium [9]. The molecular pathways for the development of these populations of cells have been enumerated by single gene knock out studies [e.g., 5,6,9,10]. The promise of a population assessment of gene expression over time offers the possibility of identifying novel genes critical to cerebellar development. By taking advantage of FANTOM5 cerebellar developmental time course analysis, we plan to identify the key regulators in cerebellar development with primary focus on cerebellar granule cells.

We used the mouse time course tissue prepared for the FANTOM5 HeliScopeCAGE dataset, to investigate the key transcriptional regulators of cerebellar development. To elucidate the genetic regulation in cerebellar development, we profiled the motif activity patterns of 534 transcription factors over 12 time points. Novel transcription factors were identified based on similar motif activity to known transcription factors critical to cerebellar development. To experimentally validate our CAGE data and bioinformatics predictions, 6 TFs novel to cerebellar development were further studied using real-time PCR experiments and in utero knock down experiments. We found that Atf4, Rfx3, and Scrt2 knock-down embryos exhibited severe cerebellar defects, suggesting these TFs are critical for cerebellar development.

## Results

### Time-course CAGE data collection and quality control

**Fig. 1:**
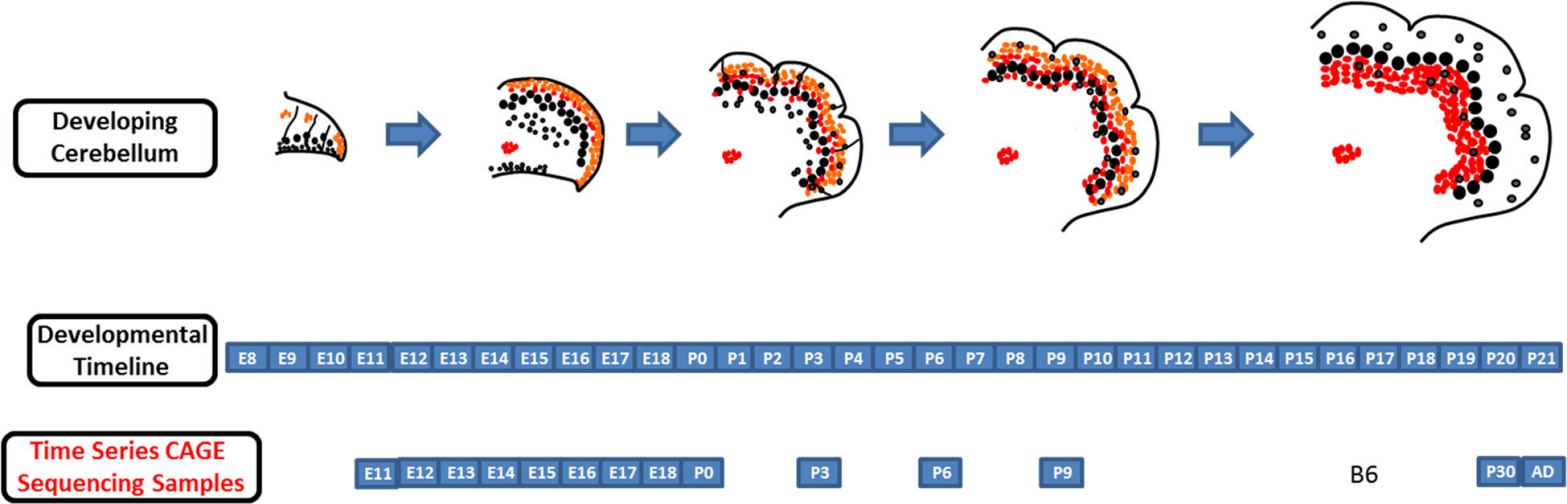
Overview of FANTOM5 HeliScopeCAGE transcriptome dataset. Timeline and schematic diagram of cerebellar development over embryonic and postnatal development and the time points selected for time series microarray data collection. Orange-colored cells – granule cells and their progenitors. Black-colored cells – interneurons and their progenitors. Red-colored cells – Purkinje cells and their progenitors. E# - Embryonic Day #; P# - Postnatal Day #

**Fig. 2:**
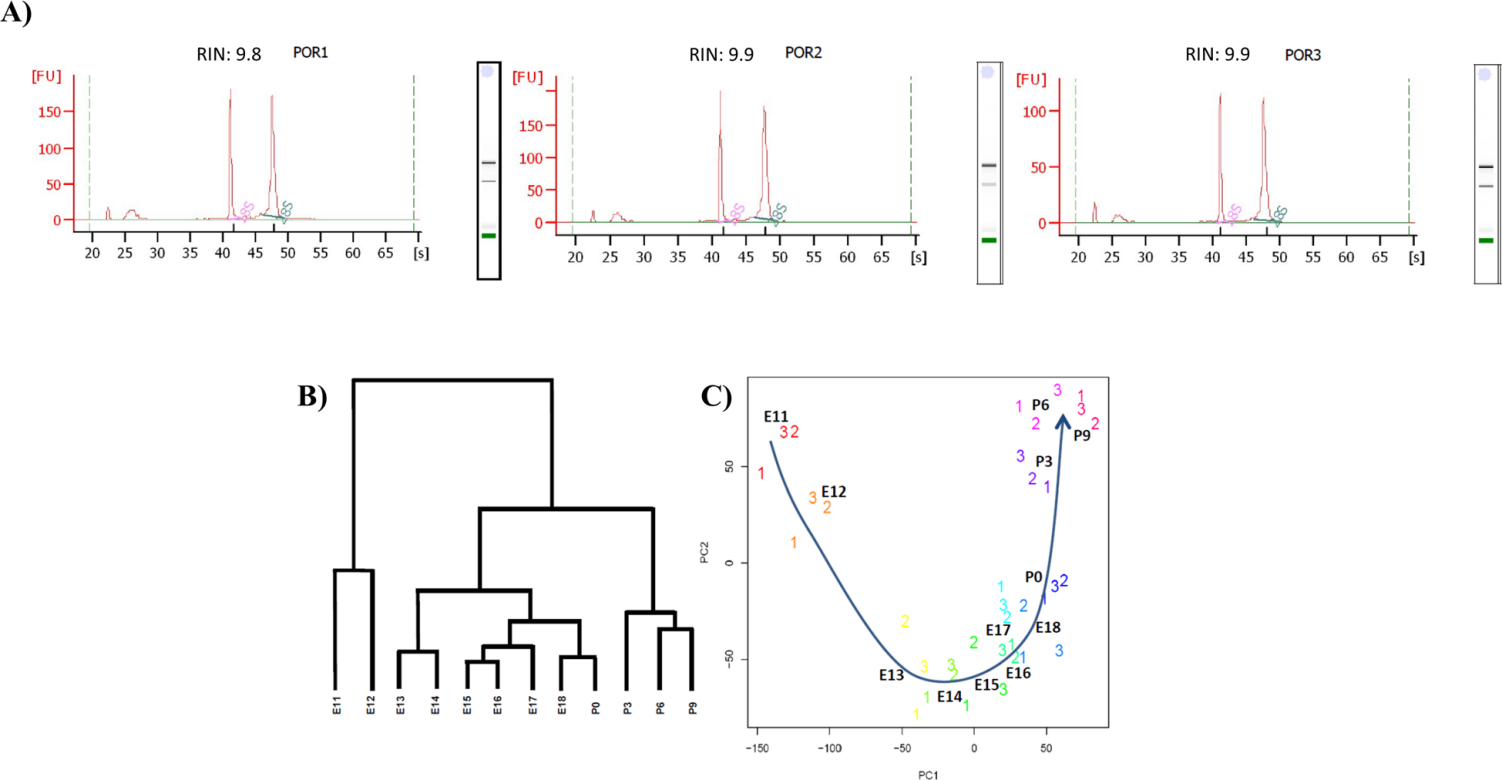
Clustering analysis of the cerebellar development time-points. **a**) Bioanalyzer graph shows the RNA integrity of our cerebellar RNA samples. The two peaks in the center represent 18S and 28S rRNA respectively and a RIN score (max of 10) is measured by aggregating the electrophoretic trace of the RNA sample. **b**) Hierarchical clustering. Hierarchical clustering of cerebellar development on time points (mean expression of three biological replicates) show three major groupings. The very early embryonic time points (E11 and E12) are the most distant from other late embryonic (E13-E18, P0) and postnatal (P3-P9) time points. **c**) Principal component analysis (PCA). PCA on cerebellar development shows a clear temporal trajectory from early embryonic E11 to postnatal time point, P9

As a part of the FANTOM5 Consortium effort to annotate the regulatory regions involved in gene expression in mammals, we have generated a time-series of whole cerebellum samples consisting of 8 embryonic and 4 postnatal time points from the C57BL/6 mouse that were subjected to CAGE experiments (Fig. 1). Each time point contained 3 biological replicates that were pooled from as many as 5–20 whole cerebellum tissues. The ages of the embryonic samples were within a narrow window using timed-pregnant matings to minimize developmental noise (see Methods). A Bioanalyzer assay of the harvested total RNA samples revealed RNA integrity numbers (RIN) of 9.6 or higher for all but two samples (see Fig. 2a, P0 Bioanalyzer results as an example).

In total, we generated 36 HeliScopeCAGE libraries (12 time points with 3 biological replicates per time point) from whole mouse cerebella from E12 to P9. There is a total of 183,903,557 tag reads (from 1,648,798 to 9,020,041 tag reads, median = 5,455,656). These reads were associated with 25,207 unique refSeq transcripts (which would produce a unique transcript) associated with 20,027 unique known refSeq genes [1]. We used the unique transcripts with distinct TSSs for our expression analyses as different promoters of a single gene may have different expression patterns leading to products that have distinct functions.

The concordance among biological replicates was examined using a sample clustering approach (Fig. 2b and c). Hierarchical clustering of the CAGE data at all 12 time points during cerebellar development showed a tight grouping of adjacent time points where the changes in early embryonic time points (E11 and E12) are distinctly separated from the expression profiles during late embryonic (E13-E18, P0) and postnatal (P3-P9) time points (Fig. 2b). A principal component analysis revealed an orderly pattern of temporal trajectory from early embryonic to postnatal time points (Fig. 2c). These results affirm the high quality of the CAGE data and the shifting of the expression profiles over the time course.

### Selection of transcription factors as potential key regulators of cerebellar development

Transcription factors have been shown to be key drivers of cerebellar development. To arrive at an experimentally tractable number of new TF candidates we performed a three-stage filtering of the 1675 known mouse TFs [10]. In stage 1 we identified the highest expressing TFs (calculated by MAX [MEAN (three bio-replicates) at each time point], see Methods) that had not been previously identified to have an involvement in cerebellar development (ie, there were no published studies indicating a given TF was involved in cerebellar development from the PubMed database). For comparison, five well documented genes that are known to be involved in cerebellar development (Atoh1, Neurod1, Pax6, Rora, Zic1) have expression levels in the range of the unknown, newly identified genes (see bottom of Table 1). As a final criterion in stage one filtering of candidates, we identified all the TFs that either had no knockout data or if a KO existed there was an attendant phenotype but no specific data on the cerebellum (http://www.informatics.jax.org/). The top 20 genes that met these criteria (see Table 1) were further filtered in a second stage of analysis. Here candidates needed to demonstrate expression in the cerebellum [using either the Allen Brain Atlas (http://www.brain-map.org/) or Eurexpress (http://www.eurexpress.org/ee/)]. Twelve of the 20 candidates in Table 1 met this criterion.

**Table 1:** The expression of 20 novel candidate transcription factors in cerebellar development (bolded genes are those examined with qRT-PCR and in utero knock-down). Five genes well known in cerebellar development with their expression levels for comparison are shown at the bottom of the table, in italics

Refseq	Gene symbol	Mean	Max	KO phenotype	Eur-express	Allen Brain
NM_010496	Id2	341	529	postnatal lethality		Y
NM_057172	Fubp1	252	469	NO KO		
NM_011865	**Pcbp1**	212	365	NO KO		Y
NM_016889	**Insm1**	74	183	perinatal and neonatal lethality	Y	
NM_001160410	**Scrt2**	80	180	NO KO	Y	
NM_001008542	**Mxi1**	90	168	progressive hyperplasia in the spleen and prostate		Y
NM_009716	**Atf4**	117	167	postnatal lethality		Y
NM_130893	Scrt1	76	161	NO KO		
NM_001103165	Pcbp2	90	147	NO KO		
NM_001024918	Rfx4	52	138	missing dorsal-mid line structure of the cortex, neonatal lethality		Y
NM_009628	Adnp	100	132	embryonic death between E8.5 and E9		
NM_010772	Maz	101	128	NO KO		Y
NM_011265	**Rfx3**	77	127	embryonic and perinatal lethality,		Y
NM_181322	Ctcf	86	120	die prior to E9.5		
NM_013780	Npas3	56	114	exhibit abnormal behavior and nervous system morphology	Y	
NM_001085492	Rere	65	88	embryonic lethality with abnormalities in neural tube development		
NM_011732	Ybx1	60	86	embryonic and perinatal lethality		
NM_001093776	Myt1l	49	85	NO KO		Y
NM_023739	Nfx1	74	84	NO KO		
NM_015753	Zeb2	41	83	embryonic death between embryonic days 9.5 and 10.5		Y
*Expression of genes known to cerebellar development*						
*NM_010894*	*Neurod1*	*299*	*1035*			
*NM_009573*	*Zic1*	*216*	*408*			
*NM_001244200*	*Pax6*	*58*	*107*			
*NM_001289916*	*Rora*	*35*	*59*			
*NM_007500*	*Atoh1*	*28*	*58*			

A third stage of analysis was conducted in parallel to the first two. In this analysis we examined all 534 TFs in the JASPAR database which contains a comprehensive collection of transcription factor binding sequences, ie, the motif matrices [11]; this analysis is described in the following section. The intersection between the two analyses and the resulting candidate set yielded the genes chosen for functional validation.

### Transcription factor motif activity during cerebellar development

Transcription factors (TFs) regulate gene expression by binding to specific DNA sequences at *cis*-regulatory regions of their target genes. As TFs bind to the DNA in a sequence-specific manner, their binding sites can be computationally modeled by motifs representing their binding preferences [12, 13]. The binding of a TF to its binding sites can result in the activation (indicating a positive motif activity) or repression (indicating a negative motif activity) of the transcription of its target, depending on the cellular context [13]. To further shed light onto the regulatory transitions observed along the time-course, we determined the motif activity of TFs as predicted by the transfactivity tool of the REDUCE suite 2 [14]. The tool used TF binding profiles from JASPAR [11] to computationally predict 534 TFs that dynamically regulate their downstream targets through binding to their promoter and enhancer regions using a multiple-linear regression approach.

**Fig. 3:**
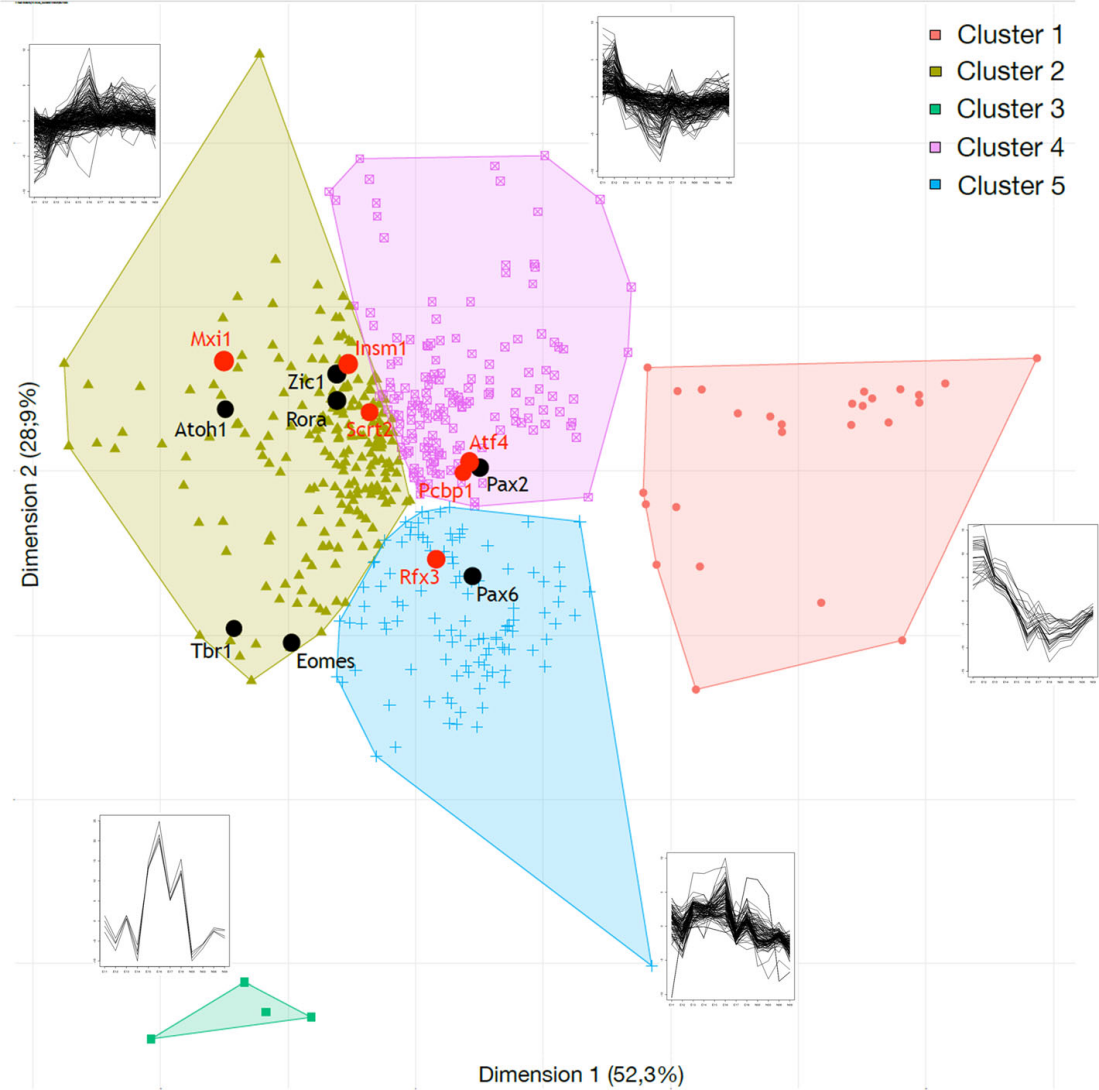
K-mean clustering of TFs based on their motif activity pattern in cerebellar development. Transcription factors are clustered based on their temporal motif activity pattern in cerebellar development. The motif activity profiles are shown beside each cluster. Genes highlighted with black dots have known functional roles during cerebellar development. Potential novel regulators with similar motif activity patterns can be identified as a neighbor to known genes. Six candidates – Atf4, Insm1, Mxi1, Pcbp1, Rfx3 and Scrt2 that were used for validation experiments, highlighted with red dots, are neighbors to known cerebellar regulators - Atoh1, Pax2, Pax6, Rora and Zic1, highlighted with black dots

In order to identify candidate TFs important for cerebellar development, we searched for TFs that showed similar motif activity pattern to known cerebellar regulators. All 534 TFs from the transfactivity analysis were partitioned by a k-means clustering according to their motif activity pattern (Fig. 3). A silhouette score analysis was carried out to determine the number of clusters (aka. The k-value) that can effectively represent the informative relations. We chose the significant k-value that produced clusters with no more than 50% of the total TFs (i.e., 267 TFs). This corresponded to k = 5 (with a silhouette score of 0.26, Additional file 1: Figure S1). Our biggest cluster (cluster 1 in Additional file 1: Figure S1) contains 163 TFs (the red cluster in Fig. 3), including key genes known to be critical to cerebellar development such as Pax6; while our smallest cluster (cluster 5 in Additional file 1: Figure S1) only contains 4 TFs (the green cluster in Fig. 3): NFATC1, NFATC2, NFATC3 and NFAT5, which all belong to the “Nuclear factor of activated T-cells” family.

**Fig. 4:**
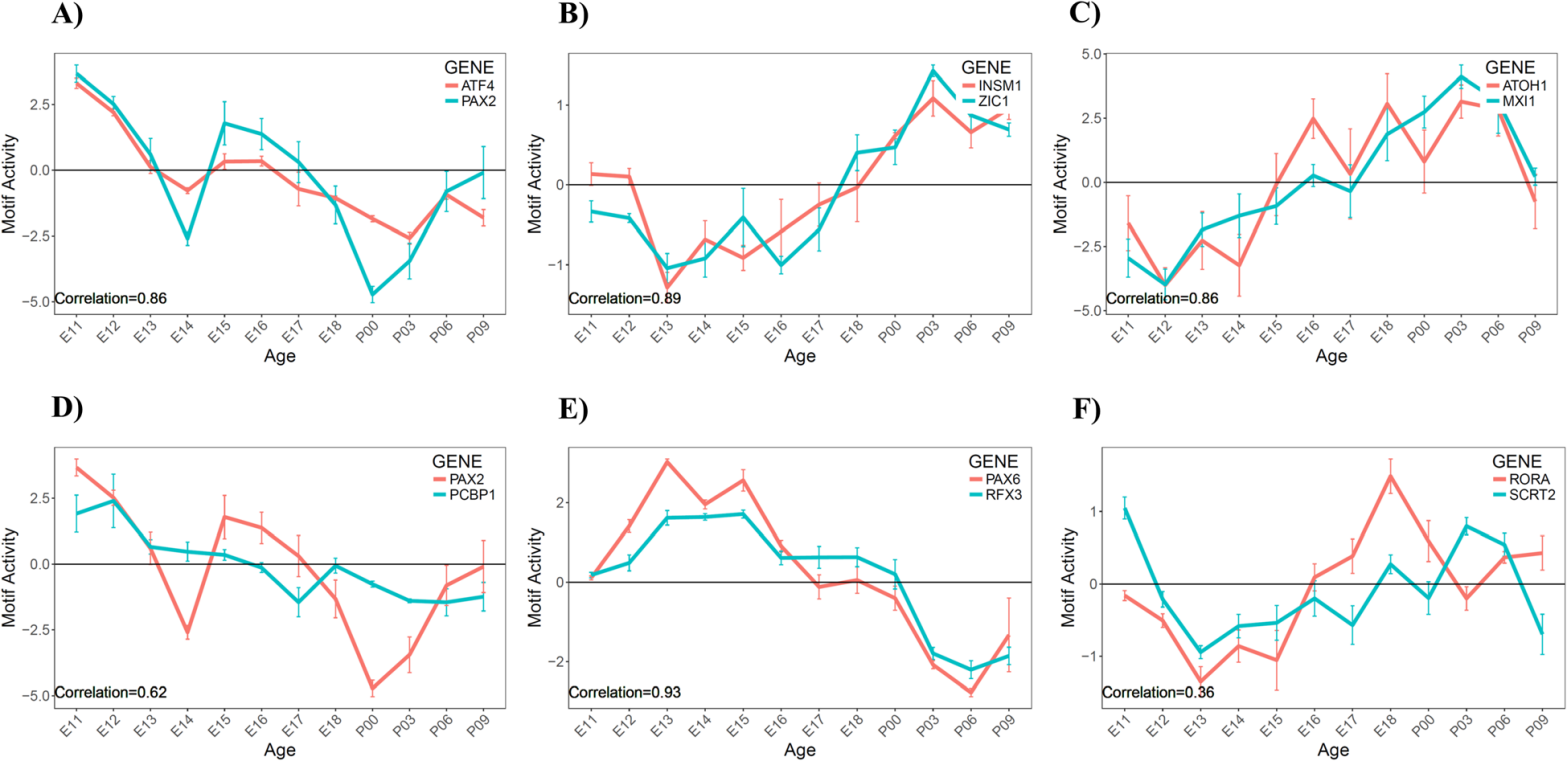
Identification of validation candidates with similar motif activity patterns to known cerebellar regulators. The motif activity (y-axis) is measured at different time points of the time series (x-axis). A positive activity indicates an overall activation of downstream targets sharing the motif; and a negative activity indicates an overall suppression of downstream targets; a motif activity of 0 indicates no change in expression between the two consecutive time points. Motif activities of known and novel TFs are shown with blue and red plots, respectively. **a**. interneuron regulator Pax2 vs. novel TF Atf4. **b**. The granule cell regulator Zic1 vs. novel TF Insm1. **c**. The granule cell regulator Atoh1 vs. novel TF Mxi1. **d**. The interneuron regulator Pax2 vs. novel TF Pcbp1. **e**. The granule cell regulator Pax6 vs. novel TF Rfx3. **f**. The Purkinje cell regulator Rora (var. 2) vs. novel TF Scrt2

We selected candidate TFs for their motif activity pattern positively correlated with known cerebellar regulators. For example, well known genes such as Pax6 and Atoh1 whose roles in cerebellar development are well-established were excluded from the list and were used as “seeds” to search novel TFs with highly correlated motif activity pattern. With a focus on the 12 TFs from our previous analysis (see above), we found that six genes novel to cerebellar development – Atf4, Insm1, Mxi1, Pcbp1, Rfx3 and Scrt2 -- are positively correlated (*p* < 0.05) with a gene known to be critical for cerebellar development. Four of these genes - Atf4, Insm1, Mxi1 and Rfx3 are highly correlated (Pearson correlation, r > 0.80) to known cerebellar regulators in motif activity pattern, which suggest that they could function as co-factors or in the same transcriptional pathways (Fig. 4): Atf4 (a neighbor to Pax2, Fig. 4a), Insm1 (a neighbor to Zic1, Fig. 4b), Mxi1 (a neighbor to Atoh1, Fig. 4c), Pcbp1 (a neighbor to Pax2, Fig. 4d), Rfx3 (a neighbor to Pax6, Fig. 4e), and Scrt2 (a neighbor to Rora var. 2, Fig. 4f). As further confirmation, we found that these six identified TFs shared similar motif activity patterns to their corresponding neighboring, known TFs – a hint that they may function in the same cell population or in the same genetic regulatory pathway (Fig. 4). These TFs were further explored as candidates using qRT-PCR and bioinformatic analyses of promoter and enhancer activity.

### Quantitative expression validation with qRT-PCR

**Fig. 5:**
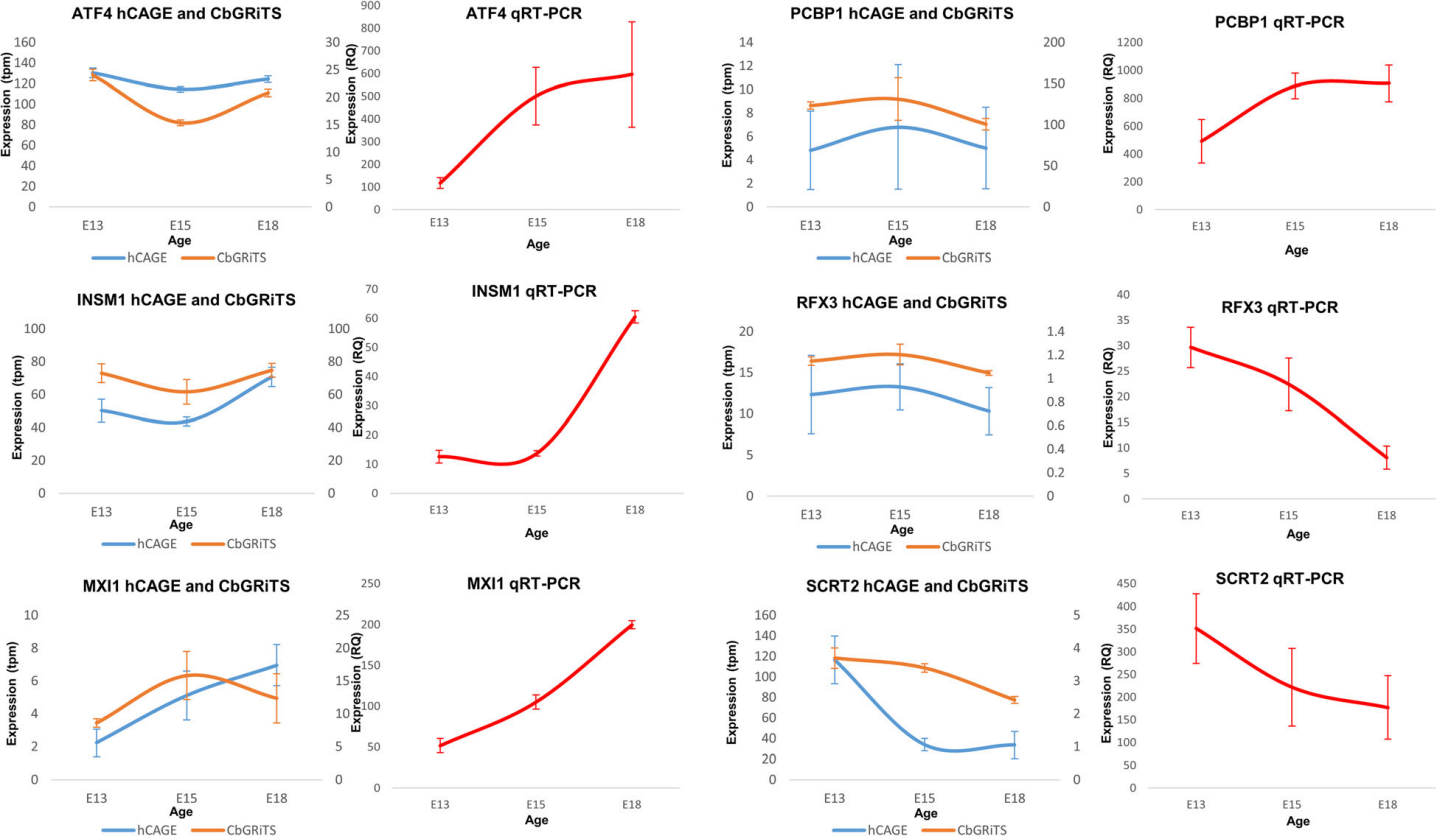
Validation of HeliScopeCAGE expression for six differentially expressed transcription factors with qRT-PCR. Quantitative real-time PCR validation for the expression of Atf4, Insm1, Mxi1, Pcbp1, Rfx3 and Scrt2. Gene expression is measured – relative quantity (RQ, quantity relative to H2O negative control) for qRT-PCR and tpm for CAGE genes were shown on y-axis. Expression at E13, E15 and E18 (x-axis) were sampled for prediction and validation groups to representative embryonic developmental stages. Expression pattern is used as indication for the accuracy of CAGE data

For quantitative validation, we performed real-time PCR on these six genes (see Fig. 5). We observed a similar expression pattern between our CAGE data (Fig. 5, bottom panel) and RT-PCR data (Fig. 5, top panel). For example, Mxi1 and Insm1 showed activated expression at E15 and E18 from the cerebellar CAGE data and their qRT-PCR showed a similar pattern; while Scrt2 and Rfx3 showed reduced expression at E15 and E18 from the cerebellar CAGE data and their qRT-PCR reflected this pattern (Fig. 5). The qRT-PCR results corroborate our cerebellar CAGE data.

### Role of promoter activity and enhancer activity in gene regulation during cerebellar development

**Fig. 6:**
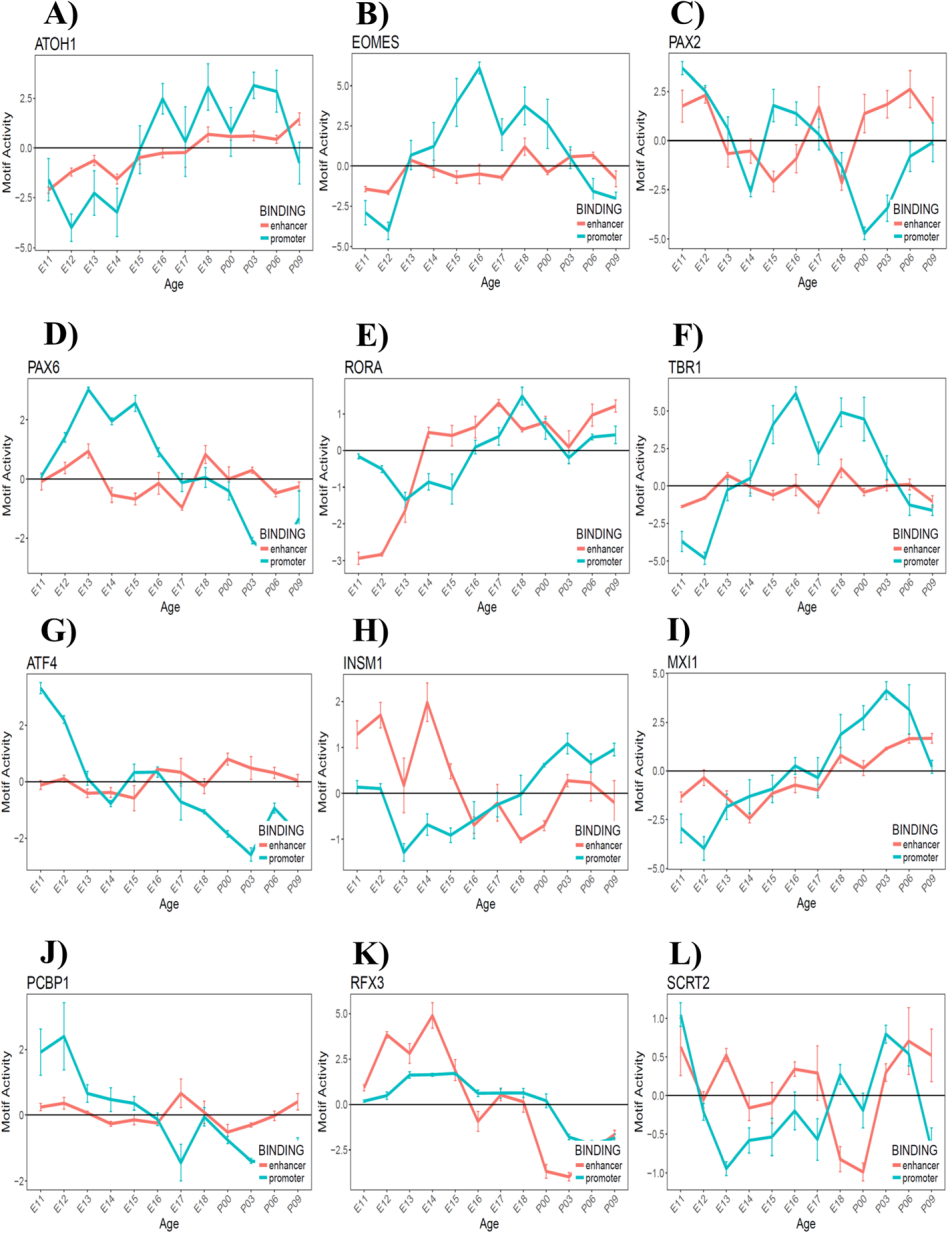
Dynamic promoter and enhancer motif activities of 12 transcription factors during cerebellar development. Six transcription factors (TFs) with a shift in motif activities of promoter (indicated by blue plots) and/or enhancer (indicated by red plots) during cerebellar development. **a**-**f**) Promoter and enhancer motif activity of 6 known cerebellar regulators: **a**) Atoh1, **b**) Eomes, **c**) Pax2, **d**) Pax6, **e**) Rora, **f**) Tbr1. **g**-**l**) Promoter and enhancer motif activity of 6 novel cerebellar regulators: **a**) Atf4, **b**) Insm1, **c**) Mxi1, **d**) Pcbp1, **e**) Rfx3, **f**) Scrt2

In addition to identify binding motifs in promoter regions, we also used the Transfactivity tool to computationally predict motif binding activity in the enhancer regions in cerebellar development. We were able to identify 252 activated motifs in the enhancer region of all genes, suggesting that these TFs can regulate their downstream targets by binding to their cis-acting enhancer regions. Figure 6 shows 12 representatives TFs which showed dynamic promoter and/or enhancer motif activities during cerebellar development: 6 TFs known to cerebellar development (Fig. 6a-f) and our newly identified candidate novel cerebellar regulators (Fig. 6g-l).

While all of the 6 known TF regulators are suggested to bind to the promoter regions of their target, Pax6 and Rora also showed enhancer motif activities that significantly deviated from 0 motif activity (Fig. 6c and e). More specifically, the granule cell transcription factor Pax6 regulates its downstream targets by binding to the promoter region, whereas its enhancer motif activity remains roughly 0 throughout development (Fig. 6d). Pax6 showed positive motif activities (which suggest an up-regulation of Pax6 downstream targets) during embryonic development when the granule cells are generated and proliferating. Interestingly, Pax6’s motif activity declines to negative values (which suggests a down-regulation of Pax6’s downstream targets) at post-natal time points when the granule cells differentiate and mature. Atoh1, known for its roles in granule cell specification, also showed little activity at enhancer regions; however, opposite to Pax6’s motif activity, Atoh1 showed a shift from negative activity (down-regulation of its targets) before E15 to positive values (up-regulation of its targets) from E16 to P6 (Fig. 6a).

Our six novel candidates for cerebellar gene regulation showed dynamic promoter motif activities, similar to the 6 known TF regulators; this is expected as the promoter motif activities are positively correlated between the known and novel TFs (shown in Fig. 4). Additionally, dynamic motif activity patterns are observed in 4 out of 6 of our novel candidate genes in the enhancer regions: Insm1, Mxi1, Rfx3 and Scrt2 (Fig. 6h, i, k and l, respectively). Atf4 and Pcbp1 do not demonstrate motif activity in the enhancer region but their promoter motif activity patterns are similar to Pax6 with an earlier activation time (E11 compared to E13 activation of Pax6; Fig. 6g and j). Mxi1 and Rfx3’s motif activity patterns in the enhancer regions are similar to their promoter counterpart (Fig. 6i and k). For Mxi1, the enhancer activity is less dynamic than the promoter activity (Fig. 6i). On the other hand, Rfx3, predominantly binds to the enhancer region of its downstream targets as its enhancer motif activity is highly dynamic compared to its promoter counterpart: it shifts from positive values before E15 to a negative value at post-natal time points (Fig. 6k).

Finally, Insm1 and Scrt2 not only showed highly dynamic enhancer motif activity patterns (Fig. 6h and l), but they also showed negative correlation between enhancer activities and their promoter counterpart. This suggests Insm1 and Scrt2’s regulation of their downstream targets is region specific – they may suppress transcription of their targets when binding to the promoter regions and activate transcription when binding to the enhancer regions (e.g. Insm1 at E14, Fig. 6h), and vice versa (e.g. Scrt2 at E18, Fig. 6l).

### Scrt2, Rfx3 and Atf4 as critical regulators of the cerebellum development

**Fig. 7:**
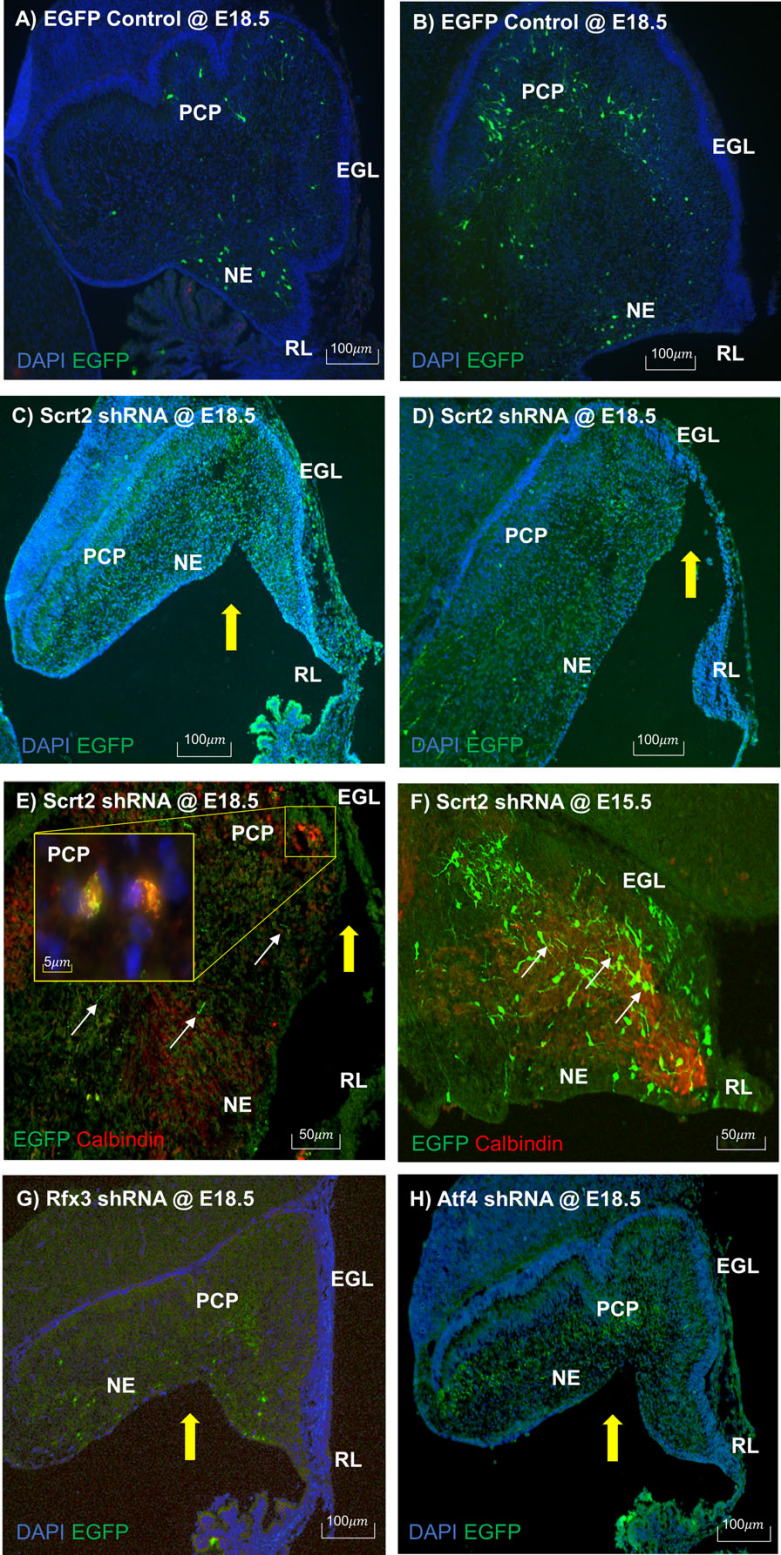
Knockdown of Scrt2, Atf4 and Rfx3 showed similar phenotypic alterations during cerebellum development. In utero transfection with shRNA against Scrt2, Atf4 and Rfx3 was performed at E12.5 and harvested at E18.5 (**a**-**d**, **f**-**h**) and E15.5 (**e**). The harvested cerebellar tissue was sectioned and immunofluorescent staining with EGFP (all) and Calbindin (**e** and **f**) antibody was performed. **a**) Control EGFP transfection – medial section. **b**) Control EGFP transfection – lateral section. **c**) Scrt2 shRNA transfection – medial section. **d**) Scrt2 shRNA transfection – lateral section. Note yellow arrows indicate location of developmental abnormality and tissue atrophy. The Scrt2 shRNA transfected cerebella seems to exhibit developmental retardation such as lack of foliation at E18.5. **e**) Double color immunofluorescent staining with EGFP and Calbindin antibody on Scrt2 shRNA transfected cerebellar tissue at E18.5Most of the EGFP+ Scrt2 shRNA transfected cells have disappeared and only their processes (punctate EGFP signals) remain. **f**) Double color immunofluorescent staining with EGFP and Calbindin antibody on Scrt2 shRNA transfected cerebellar tissue at E15.5. Many of the EGFP+ Scrt2 shRNA transfected cells are positive for calbindin staining as well, indicated by thin white arrows. **g**) Rfx3 shRNA transfection – lateral section. H) Atf4 shRNA transfection – medial section . In utero transfection against Rfx3 (**g**) and Atf4 (**h**) shRNA showed a similar, but less dramatic phenotype of tissue atrophy and developmental delay as in the Scrt2 knockdown. EGL – External granular layer, NE – Neuroepithelium, PCP – Purkinje cell plate, RL – Rhombic lip

We made shRNA knockdown constructs for the 6 genes that emerged from the filtering work described above and tested the effect of knockdown on early cerebellar development. We injected shRNA constructs into the IVth ventricle, electroporated with EGFP plasmids at E12 and processed the brain for any phenotypes at E18 (6 days later). Three of the 6 candidates, Atf4, Rfx3 and Scrt2, showed morphological perturbations of the developing cerebellum (Fig. 7). Scrt2 showed the most striking phenotype with tissue atrophy and developmental delay compared to the control injected cerebellum (Fig. 7a-d). At the gross level, the Scrt2-knockdown cerebellum size was dramatically reduced with the apparent elimination of a region of the cerebellum that provides continuity between the neuroepithelium and the rhombic lip. Foliation had not commenced in either the medial or lateral cerebellum of the Scrt2 knockdown cerebella compared to the clear initiation of foliation in both of those regions in the control transfected brains. In the Scrt2 knockdown, there were very few live EGFP positive cells at E18.5 while there were always labeled cells in the controls (compare Fig. 7c-d to a-b). The only EGFP positivity in the Scrt2 knockdown was found in processes, presumed to be dendrites or axons of degenerated cells; indicative of successful transfection.

To investigate what became of the Scrt2 shRNA transfected cells, we harvested embryos at an earlier time point, three days after transfection, at E15.5. Morphologically there were no noticeable differences between the control and Scrt2 shRNA transfected group at E15.5 (Fig. 7f; 3 days after transfection). We co-labeled with BrdU to determine if at this time there were marked perturbations in cell proliferation and found none (see Additional file 2: Figure S2). In addition, we did not observe an increase in anti-activated Caspase3-positive cells at E15.5 in the Scrt2 knock down cerebellum (see Additional file 2: Figure S2). This indicates that there was an extensive loss of cells between E15.5 and E18.5 following transfection at E12.5. Of those cells that are EGFP+, they also stain positive for Calbindin, suggesting that either the surviving transfected cells are Purkinje cells, or that these cells are the products of a single hit with EGFP with no gene knockdown (Fig. 7g-i).

**Fig. 8:**
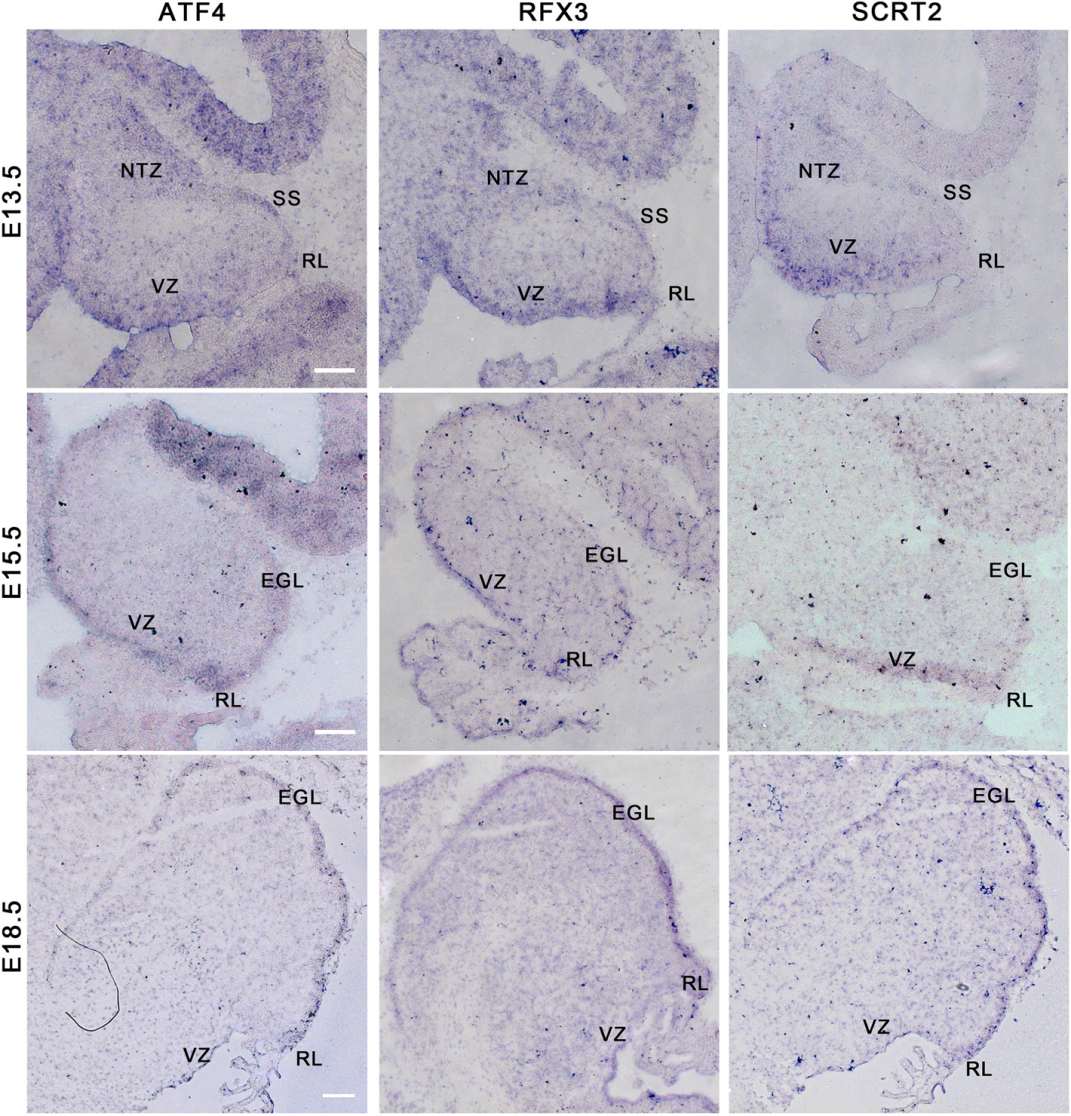
Expression of Atf4, Rfx3 and Scrt2 in the developing cerebellum demonstrated by in-situ hybridization. (Top row) At E13.5, all three genes are prominently expressed in the neuroepithelial ventricular zone (VZ). In addition, Atf4 and Rfx3 expression is observed in the subpial stream (SS) and nuclear transitory zone (NTZ). Rfx3 is also expressed in the rhombic lip (RL). (Middle row) At E15.5, expression of all three genes remain prominent at the VZ. Expression of all three genes is now seen also in the external geminal layer (EGL) that contain the granule cell precursors. (Bottom row) At E18.5, expression in the VZ has ceased for Atf4 and Scrt2, while Rfx3 expression is still observed in the VZ. All three genes are prominently expressed in the EGL at this age

The knockdown of Rfx3 and Atf4 showed similar phenotypes to the Scrt2 knockdown with tissue atrophy in the neuroepithelium and developmental delay (Fig. 7g and h). In order to explore these results, we examined the expression of these three genes using in situ hybridization. As predicted from the knockdown results, the expression pattern of these three genes were almost identical. During early development each gene was specifically expressed in the mitotically active regions of the cerebellum (see Fig. 8). Furthermore, we identified potential targets of these three genes using the Transfactivity analysis described above (see Additional file 3: Table S1). However, the atrophy observed in Scrt2 was far more extensive than seen with knockdown of Rfx3 and Atf4. The Atf4 knockdown had the mildest phenotype among the knockdown of the three transcription factors. It retained more EGFP positive cells than the other two gene knockdowns at E18. Furthermore, there were obvious signs of foliation. The difference in phenotypic outcomes could be due to differences in the downstream targets of these three genes which had almost no overlap.

## Discussion

### Overview of the FANTOM5 cerebellar time course

The availability of large-scale CAGE data produced by the FANTOM5 consortium has been shown to provide an unprecedented opportunity to highlight the precise location of TSSs and the transcriptional events driving cellular differentiation and response to stimuli [1, 15]. Here, we generated mouse cerebellar CAGE data from 12 time-points to predict master regulators of cerebellar development. Out of the 33 FANTOM5 time courses (including 19 human and 14 mouse time courses), our cerebellar development time course is one of the two developmental time courses [1]. While the other FANTOM5 developmental time course – the visual cortex – consisted of mutant mouse tissues from 3 postnatal time points, our cerebellar time course is unique in that it covered normal development of a CNS region from embryonic to postnatal time points [1]. With the cerebellar time course, we first validated the quality of the data through orthogonal experiments and bioinformatics analyses. We hypothesized that TFs might act as key regulators in the development of the cerebellum as highlighted by our differential expression and TF binding motif bioinformatics studies. Finally, we experimentally validated that the TFs Scrt2, Rfx3, and Atf4 are important for cerebellar development since knock-downs introduced phenotypic disturbances.

### Time course data reveal the expression landscape of cerebellar development transcriptome

Technologies that provide a genome-wide assessment of gene expression have yielded many insights into development. In particular, microarray technology has been applied to the analysis of gene expression during cerebellar development. Sato et al. have generated the Cerebellar Development Transcriptome Database (CDT-DB) sampling eight time points focused on postnatal cerebellum development (E18, P0, P3, P7, P12, P15, P21 and P56) using the Affymetrix microarray platform [16, 17]. This resource-rich database combined the microarray data with in situ hybridization, GeneChip, and RT-PCR data, which were integrated into a web-based knowledge resource, with links to relevant information at various other websites.

A second cerebellar transcriptome database, constructed by Ha et al., is Cerebellar Gene Regulation in Time and Space (CbGRiTS [18]). CbGRiTS contains over 300 Illumina microarrays that are derived from 12 developmental time points primarily focused on embryonic times (the same time points as assessed in the current study) from the C57Bl/6 J and DBA/2 J lines of mice. In addition, CbGRiTS includes transcriptome data from fourteen recombinant inbred mice constructed between B6 and D2 and three mutant lines of mice whose mutant genes are known to target cerebellar granule cells (Atoh1, Pax6, and the meander tail mutant [18]). CbGRiTS is an exceptional tool to investigate gene expression patterns over time and it provides several bioinformatic algorithms which allow the generation of genetic regulatory networks in cerebellar development. Furthermore, after the exploration of a given gene in the CbGRiTS microarray database, one can link-out directly to the gene’s corresponding pages in other anatomical databases [18].

While CDT-DB and CbGRiTS were among the largest transcriptome databases focusing on a single brain structure during mammalian brain development, bioinformatics analysis and identification of master gene regulators of brain development have been challenging due to lack of methods linking transcriptional activity with specific promoter use, an ability to compare multiple brain regions in the same platform, and an overall validation of these microarray-based datasets. The FANTOM5 HeliScopeCAGE data, utilizing the next generation sequencing technology, allows resolution of these shortcomings. This cross-validation between datasets provides a powerful tool, combined with bioinformatics analyses, to shed light into key transcriptional events and to highlight potentially new key master regulators in cerebellar development.

While the FANTOM5 database [1] (http://fantom.gsc.riken.jp/zenbu/) has a collection of many mouse tissues that permits one to determine gene expression tissue specificity, we did not use that as a criterion in the present studies. Three of the 6 candidate genes (Atf4, Mxi1 and Pcbp1) did not show such tissue specificity. In fact Atf4, which did not show cerebellar specific expression, had a phenotype when knocked down; on the other hand, Insm1, which has exquisite cerebellar specificity, did not have a phenotype when knocked down.

Several developmental time series transcriptome datasets have been generated from different regions of the mammalian brain, such as cerebral cortex [19–22], hippocampus [23] and hypothalamus [24]. These datasets not only provide us the transcriptional landscape of different brain regions, neurodevelopmental processes, and potential causative factors for complex neurodevelopmental disorders, but can also be used to explore brain region-specific ontologies, cellular make-ups, and neurological processes. Interestingly, when compared with other brain regions such as the neocortex and hippocampus, the cerebellum is most transcriptionally distinctive [25]. In the neocortex, sub-regional transcriptional differences were observed, but less robust than the differences between brain regions (i.e. the neocortex vs. the cerebellum) [25, 26]. NB—some genes shared in ctx and cb and how they are involved, maybe in similar cascades but in different processes – examples are Pax6, Hes genes, and NFI genes … Furthermore, temporal analyses of the human time series datasets revealed a large number of genes with dynamic and/or developmental stage-specific expressions; for example, it is found that the differential gene expression during prenatal and early postnatal development accounts for more than 60% of the total gene expression variation – more than the expression changes over several decades of adulthood [25]. These transcriptome datasets revealed the role of spatial and temporal genetic regulation during development that leads to a complex and dynamically regulated regional and sub-regional transcriptional landscape in human brain.

These transcriptome datasets also revealed convergent gene expression across different brain regions [25, 27, 28]. Gene clusters with specific developmental roles can be co-regulated across brain regions and time stages that lead to the activation of specific biological processes [25]. For example, Kang et al., found that a gene cluster enriched for neuronal specification is highly expressed early, during embryonic development, while a gene cluster enriched for synaptic function and ion channels is activated in late embryonic and postnatal time stages – a common theme shared by two distinct brain regions, the cerebral cortex and the cerebellum [25]. The co-expression of such gene clusters in different brain regions can lead to analyses that identify regulators and construct transcription networks that would play important and fundamental roles in various neuronal developmental processes such as cell proliferation, migration, differentiation, and synapse formation.

### Transcriptional landscape in cerebellar development

The hierarchical clustering analysis (HCA), which reveals the overall similarity and differences of expression profiles between samples, of all 12 developmental time points revealed a transcriptional landscape consisting of 3 stages of distinct expression in cerebellar development: an early embryonic stage consisting E12-E13, a late embryonic stage consisting E14-P0 and a postnatal stage consisting P3-P9. The trajectory observed in the PCA plot (Fig. 2c) reveals the same transcriptional landscape. Of interest, HCA of CbGRiTS data also showed a similar pattern with slightly different boundary time points - the late embryonic time stage consisted of E15-E19 in CbGRiTS instead of E14-P0 revealed by this study. We can hypothesize that key events in cerebellar development occur around E13, as well as and around the time of birth.

Indeed, E13 is the time point when cerebellar granule cells emigrate from the rhombic lip to form the external granule layer which is the major site of pre-granule cell proliferation [27–29]. Thus, the current CAGE-based time course dataset provides an excellent resource to investigate the molecular changes that might precede, coincide, or be proximal with time-specific cerebellar developmental events. The current dataset also can be used as a valuable reference point to gauge the tissue and cell-type specific expression of transcriptional networks in the developing brain.

### Dynamic motif activity of key cerebellar regulators

The Transfactivity tool can measure the regulatory activity of TFs at promoter and enhancer regions of genes during the cerebellar time course [12]. Transfactivity analysis (also called motif activity analysis) infers transcription factor activity through analysis of the expression patterns of elements (genes/promoters/enhancers) putatively controlled by the transcription factors [30]. This is accomplished by locating the elements carrying proximal binding sites for the factors, and then model the expression of the elements as a function of a transcription factor activity that is fitted to the expression data [30]. In our CAGE dataset, the motif activity patterns often do not match the expression patterns of the corresponding transcription factors, this could be explained that transcription factor activity is dependent not only on expression levels, but also on other processes like post-translational modifications (e.g. phosphorylation), sub-cellular localization (e.g. nuclear translocation) and interaction with other molecules (e.g. protein-protein interaction with co-factors) [30]. While these processes are difficult to measure directly in a high throughput way, Transfactivity analysis allows indirect measurement of TF motif activities through expression analysis of the predicted targets of the transcription factors.

Through Transfactivity analysis, we found that the downstream targets of Pax6, a key regulator in cerebellar granule cells, were up-regulated during embryonic development when the granule cells are generated and proliferative [31, 32]. In contrast, the same set of genes regulated by Pax6 were down-regulated at post-natal time points when granule cells differentiate and mature [17, 33]. Atoh1 (aka Math1) is another key regulator of granule cell development [3, 6]. We observed an interesting transition from down-regulation of Atoh1-associated target expression before E15, to up-regulation from E16 to P6. Notably, the motif activity of Pax6 and Atoh1 are inversely correlated throughout cerebellar development, which suggests that they may function in a coordinated gene regulatory pathway, where one might serve as a functional suppressor of the other.

### Novel key regulators in cerebellar development

The FANTOM5 data led, using filtering steps, to the identification of a manageable set of six candidate genes to explore from a larger set. Interestingly, three of these six genes (Atf4, Rfx3, and Scrt2), when knocked down, yielded a perturbed phenotype. While all three TFs showed similar phenotypes, Scrt2 showed the most severe one. Compared to EGFP control, Scrt2 knockdown cerebellum showed marked neuroepithelium and EGL atrophy, lack of foliation at E18.5 and developmental delay. Furthermore, there were virtually no live Scrt2 shRNA (+) and EGFP (+) cells at E18.5. This suggests that neuronal cell death may be the mechanism of tissue atrophy and developmental delay. Co-staining of transfected embryos with Calbindin, at E15.5 and E18.5, revealed double labeled cells suggesting that the major portion of the Scrt2 shRNA (+) and EGFP (+) cells are in fact Purkinje cells. Premature death of these Scrt2 shRNA (+) and EGFP (+)/Calbindin (+) Purkinje cells could explain marked neuroepithelium and EGL atrophy, lack of foliation and developmental delay due to lack or reduced production of critical neurotrophic factors (such as Shh [34] and Bdnf [35]) that are known to be emitted by Purkinje cells. As Atf4 and Rfx3 showed similar phenotype as Scrt2, but in a milder form, these 3 TFs may play a role in the same signaling pathway in neural development. To further explore this possibility we identified predicted downstream targets of these genes using Transfactivity analysis. Although no common target emerged from this analysis, their de facto similar patterns of expression may suggest their engagement in similar biological processes. (see Peter GO analysis, if anything presents itself).

## Conclusion

The current study uses state-of-the-art genomics technology data and detailed understandings of brain development toward the identification of regulators of cerebellar development. The FANTOM5 cerebellum time series CAGE data set is an accessible, high-quality transcriptome database for functional investigation of gene regulatory networks in cerebellar development.

## Methods

### Sample preparation

This research was performed with ethics approval from the Canadian Council on Animal Care and research conducted in accordance with protocol A12–0190. C57BL/6 J mice were purchased from JAX laboratory and bred in a room with 12/12 h light/dark controlled environment. Embryos were obtained from timed pregnant females at midnight of the day when a vaginal plug was detected; this was considered embryonic day 0 (E0). Pregnant females were cervically dislocated and embryos were harvested from the uterus. The cerebellum was isolated from each embryo, pooled with littermates of like genotype, and snap-frozen in liquid nitrogen. 3–4 replicate pools of 3–10 whole cerebella samples were collected from 12 time points across cerebellar development (embryonic days 11–18 at 24 h intervals and every 72 h until postnatal day 9).

### Sample quality

Bioanalyzer analysis was performed to check RNA quality. All RNA samples used for the time series achieved high RNA Integrity (RIN) Score. 34 out of 36 samples had RIN score of 9.7 or higher (10 being the best). The hierarchical clustering analysis (HCA) and principal component analysis (PCA) were perform according to methodology established previously [15].

### Selection of transcription factors as potential key regulators of cerebellar development

To select potential key regulators, the first filtering step of the 1675 known mouse TFs [10] was based on expression level. To extract highly expressed TFs, we ranked all 1675 TFs based on their expression level measured by HeliScopeCAGE. For each TF, expression at each time point was calculated as the mean of three bio-replicates. Then, we calculated the maximum expression of the TF by the highest expression across the full set of 12 time points. The formula for calculation a TF’s maximum expression was calculated as:

Max expression = MAX [MEAN (three bio-replicates)].

Then, all 1675 TFs were ranked by their maximum expression and the top 20 TFs were used for following analyses for potential validation targets.

### Calculating transcription factor activity

The activity of the TFs at promoters and enhancers was determined using the Transfactivity tool from the REDUCE Suite, version 2.0 [36]. This statistical method, based on multiple linear regressions, characterizes the variations of a dependent variable, corresponding to the binding of TFs at cis-regulatory elements, with respect to a set of independent variables corresponding to target genes.

For each gene, a DNA sequence of 400 bp was used for computing binding affinities of promoters and enhancers. The sequence used for promoters started 300 bp upstream and ended 100 bp downstream of the dominant peak of the.

promoter (the position within the promoter with the highest expression.

value across FANTOM5 samples). The sequence used for enhancers centered.

around the enhancer region midpoint, extending 200 bases upstream and downstream.

The prediction of the affinities between the TFs and the regulated genes is carried out by the AffinityProfile, which is integrated into the Transfactivity program, using TF binding profiles from JASPAR [11, 37]. At the time we accessed the JASPAR database, it had 534 TF motif matrices which represented about 1/3 of the 1675 known TFs [11]. For the TFs without available motif matrices, we dealt with them in one of two ways: 1) if they belonged to a gene family, we estimated their motif activities from another member of its family that had computed motif matrices, 2) the TFs that did not belong to any gene family with motif matrices were excluded from our motif analyses.

Therefore, this approach predicts the regulatory activity of TFs at the level of promoters and enhancers over time by combining TF binding predictions at these elements with gene expression captured by CAGE.

### Analysis of motif activity profiles for transcription factors at the level of the promoter

In order to define the binding behavior of the transcription factor at the promoter in cerebellar tissue, the pattern data were partitioned by k-means clustering. It is an unsupervised learning that groups the transcription factors according to their activity profile. This heuristic algorithm uses the centroid principle which is the geometric center of a cluster and will minimize the distance between a point and a centroid to assign this point to a cluster. With this approach it is necessary to define the number k, and therefore the cluster number that we will attribute to our data.

To find the k value that is a best fit with our data set, a silhouette analysis was carried out to determine the inter-clusters distances and thus to know if our clusters are informative relative to each other. A simulation silhouette analysis for several k values was conducted, and the most significant value corresponded to k = 5 for an average silhouette score of 0.26. According to this outcome, it is possible to realize the k-means clustering with k equal to 5 and group the TFs according to their profile activity. Results are presented in Fig. 5. These k-means clustering were validated through a descriptive approach and by calculating the Spearman correlation coefficient between the points belonging to the same group. Figure 3 shows all the activity profiles of the transcription factors for each cluster. It can be seen here that the activity profiles of the transcription factors from each group are similar. Through this approach, we have mapped the transcription factors according to their co-activity and indicate how the regulation of genes behaves during the formation of the cerebellum.

### qRT-PCR for confirmation of expression profiles

Cerebellar tissue was collected from the embryos and total cellular RNA was collected (TRIzol reagent, Thermo Scientific). Subsequently, cDNA was synthesized using oligo dT primers following the manufacturers protocol (High Capacity cDNA Reverse Transcription Kit; Applied Biosystems). Three biological replicates were analyzed for each of the 6 target genes at three time points (E13, E15, E18) using Applied Biosystems Fast SYBR Green Master Mix reagent and Applied Biosystems 7500 Real-time PCR system (see Table 2 for the list of primers for the 6 target genes). PCR conditions were: 95 °C for 20s, 40 cycles of 95 °C for 3 s, and 60 °C for 30s followed by 95 °C for 15 s, 60 °C for 1 min, 95 °C for 15 s and 60 °C for 15 s. Amplification of GAPDH and 18 s rRNA were used as reference samples to normalize the relative amounts of cDNA between experiments. Expression profiles for each gene were calculated using the average relative quantity of the sample at each of the three time points.

**Table 2:** The list of primers used for quantification of six target genes with qRT-PCR

Gene Name	Forward Primer	Reverse Primer
Scrt2	ACTCAGACCTCCTTCCCCTC	CCCCTCCGAAACCCTAGAGA
Rfx3	CCTGATCCGGCTGCTCTATG	TCGGTGTCTCTCCTGTCACT
Atf4	TCGGCCCAAACCTTATGACC	TGGCTGCTGTCTTGTTTTGC
Pcpb1	ACGGAAAGGAAGTAGGCAGC	CCCTCCGAGATGTTGATCCG
Insm1	TCCCCTACTCCCATTCCAGG	GGAGTCACAGCGAGAAGACC
Mxi1	CAAACTCTCCTTCGCGTCCT	TTGAGAGCCGGTGTTGACTC

### In situ hybridization to visualize gene expression

Sense and antisense riboprobes corresponding to the cDNA fragment were synthesized and labeled with digoxygenin (DIG)-UTP. A cDNA library was obtained from C57BL6/J E15.5 mouse brain using a cDNA synthesis kit (Invitrogen). Then the corresponding cDNA of each gene was produced with this cDNA library, using the gene specific forward and reverse primers (see above). Each of the resultant cDNAs was cloned into the pGEM-T vector (A3600, Promega) for the generation of cDNA templates. cDNA templates for the sense and antisense riboprobes is specifically made using the primers M13F: GTTTTCCCAGTCACGAC or M13R: CAGGAAACAGCTATGAC and the gene-specific forward or reverse primers (Table 3). Riboprobes are produced using SP6 or T7 RNA polymerase (#EP0133 and #EP0111, Thermoscientific, respectively) with the corresponding cDNA templates. The resultant riboprobes were precipitated using 5 M ammonium acetate and 100% EtOH in RNase-free environment. Riboprobes were denatured at 72 °C for 10 min, and incubated on ice for 5 min, then mixed with ULTRAhyb hybridization buffer (AM8670, Applied Biosystems) preheated at 68 °C. Prior to hybridization, sections were acetylated with acetic anhydride in 0.1 M triethanolamine at pH 8.0 and dehydrated with graded concentrations of RNase-free ethanol. Sections prepared for histology (see below) were first incubated with ULTRAhyb hybridization buffer at 68 °C in a humid chamber for 1.5 h, then replaced with riboprobe in ULTRAhyb hybridization buffer at 68 °C overnight. After hybridization, the slides were rinsed with descending concentrations of salt: 4x SSC, 2x SSC, 1x SSC and 0.5x SSC at 55 **°**C, and then incubated with an anti-Dig antibody (11,093,274,910, Roche) for 2 h at room temperature. The slides were washed with maleic buffer, followed by reaction buffer, then the slides were colorized with NTP/BICP (11,681,451,001, Roche). Following colorization, the slides were rinsed with 0.1 M PB, then post-fixed in 4% paraformaldehyde, and washed with distilled water. The slides were dehydrated with graded concentrations of ethanol and xylenes. Glass coverslips were applied to the slide.

**Table 3:** The list of primers used for in situ hybridization of three target genes

Gene Name	Forward Primer	Reverse Primer
Atf4	AATCCAGCAAAGCCCCACAA	ACAAGCACAAAGCACCTGACT
Rfx3	CAGTGTTGTGCTCAGATACGTC	ATCGATGATGGAGATGAGCAGG
Scrt2	CCTCTGCCTTTGCTAGAGAACA	AATACTGCTGAAGGTGGGGAAG

### Design of shRNA and in utero transfection

Sequences of candidate shRNA were selected from each of the six genes (Atf4, Insm1, Mxl1, Pcpb1, Rfx3 and Scrt2) based on an open source algorithm at Genscript (Table 4). Designed sequences were chemically synthesized as two complementary DNA oligonucleotides, annealed and ligated to the mU6pro vector. Each construct was verified by sequencing. An in utero transfection technique was performed at E12.5 with designed shRNA construct plasmids that were co-introduced with mU6pro vector for EGFP reporter expression into the space of the IVth ventricle using an in utero electroporation method described previously [38]. We used the Vevo 770 High-Resolution In Vivo Micro-Imaging System from VisualSonics for injection. Embryos were removed at 3 and 6 days post-transfection for histological processing using immunohistochemistry and in situ hybridization. For the three genes with cerebellar developmental defects (Atf4, Rfx3 and Scrt2), we collected 8 Atf4-transfected embryos out of 24 injections at 6 days post-infection, 7 Rfx3-transfected embryos out of 23 injections at 6 days post-infection and 11 Scrt2-transfected embryos out of 24 injections divided amongst the 3 and 6 day timepoints for phenotypic analyses.

**Table 4:** The list of primers used for shRNA Construction

Gene Name	Forward Primer	Reverse Primer
Scrt2	TTTGGAAGGTCAAACTTGACACATCAGTCTGTGTCAAGTTTGACCTTCTTTTT	CTAGAAAAAGAAGGTCAAACTTGACACAGACTGATGTGTCAAGTTTGACCTTC
Rfx3	TTTGGCAAAGCTGATAACTCTGTTCAGTCACAGAGTTATCAGCTTTGCTTTTT	CTAGAAAAAGCAAAGCTGATAACTCTGTGACTGAACAGAGTTATCAGCTTTGC
Atf4	TTTGGACAGCAGCCACTAGGTACTCAGTCGTACCTAGTGGCTGCTGTCTTTTT	CTAGAAAAAGACAGCAGCCACTAGGTACGACTGAGTACCTAGTGGCTGCTGTC
Pcpb1	TTTGGCGGACTCAACGTGACTCTTCAGTCAGAGTCACGTTGAGTCCGCTTTTT	CTAGAAAAAGCGGACTCAACGTGACTCTGACTGAAGAGTCACGTTGAGTCCGC
Insm1	TTTGCTGTGCCTTCACTCGGAGATCAGTCTCTCCGAGTGAAGGCACAGTTTTT	CTAGAAAAACTGTGCCTTCACTCGGAGAGACTGATCTCCGAGTGAAGGCACAG
Mxi1	TTTGGAACCGACGAGCTCACCTGTCAGTCCAGGTGAGCTCGTCGGTTCTTTTT	CTAGAAAAAGAACCGACGAGCTCACCTGGACTGACAGGTGAGCTCGTCGGTTC

### Immunofluorescence for in utero knockdown phenotypic analysis

Whole cerebellar tissue was isolated from embryos and kept in 4 °C 0.1 M PBS. The tissue was then embedded in Optimal Cutting Temperature (OCT) compound and sectioned at 16 μm. Samples were washed in 0.1 M PBS for 2 × 5 min followed by 0.1 M PBS-T for 5 minutes. After washing, samples were permeabilized and blocked using a blocking buffer (0.1 M PBS, 10% normal goat serum, 0.3% BSA, 0.02% NaN_3_). Samples were then incubated with a 1:200 to 1:500 dilution of primary antibodies overnight at 4 °C, while control slides were incubated with blocking buffer. Antibodies were used to demonstrate the efficacy of knockdown (anti-Scrt2 and –Atf4), successfully transfected cells (anti-GFP), cells undergoing cell division [timed pregnant females were injected intraperitoneally with BrdU (Sigma, B5002; 50 μg/g body weight) 1 h before the collection of embryos], and cell death (anti-activated Caspase-3). Following primary antibody incubation, all samples were washed with 0.1 M PBS-T for 3 × 10 minutes. Samples were then incubated with a 1:200 dilution of secondary antibody and 1:1000 dilution of DAPI for 1 h followed by 3 × 10 minute washes of 0.1 M PB and 1 × 10 minute wash of 0.01 M PB. Samples were then treated with FluorSave (EMD Millipore) and stored at 4 °C. Samples were visualized using an inverted Axio Observer A1 microscope.

The primary antibodies used for immunofluorescence were: anti-Scrt2 (sc-85,910, Santa Cruz Biotechnology), anti-Atf4 (sc-7583, Santa Cruz Biotechnology), anti-Rfx3 (sc-10,662, Santa Cruz Biotechnology), anti-eGFP (ab290, Abcam Inc), rat anti-BrdU (Abcam, Ab6326, RRID:AB_305426), and rabbit-anti-active Caspase-3 (Abcam, Ab13847, RRID:AB_443014). Secondary antibodies used were Goat anti-Chicken Alexa Fluor 488 (A11039, Molecular Probes), Goat anti-Rabbit Alexa Fluor 594 (A31631, Molecular Probes), and Donkey anti-Goat Alexa Fluor 594 (90,436, Jackson ImmunoResearch Lab. Inc.).

## Additional files

Additional file 1: **Figure S1.** Silhouette plot for k = 5. Y-axis gives information for silhouette score (Si) and each point on the x-axis is a TF motif. The average silhouette score is represented by the red dotted line and is set at 0.26. (TIF 3670 kb)

Additional file 2: **Figure S2.** The knockdown (KD) of SCRT2 in the developing cerebellum has no influence on cell proliferation or programmed cell death. Co-label of BrdU (red) and EGFP (green) in E15.5 (a) SCRT2 knockdown and (b) control cerebellum. (c) Quantitative analysis of cell proliferation in control and SCRT2 knockdown cerebella. The percentage of BrdU-positive cells of all EGFP-positive cells between control and SCRT2 knockdown cerebella is insignificant. Co-label of active Caspase-3 (red) and EGFP (green) in E15.5 SCRT2 knockdown (d) and control (e) cerebellum. (TIF 3490 kb)

Additional file 3: **Table S1.** The predicted targets of the three genes with a knockdown phenotype, from Transfactivity analysis, are shown in the table. In column B is the location of the target and in Column C is the identification of the predicted downstream gene. (DOCX 16 kb)

## Abbreviations

CAGE: Cap Analysis of Gene Expression
E#: Embryonic day #
EGL: External Granular Layer
FANTOM5: Functional Annotation of Mammalian Genome Phase 5
HeliScopeCAGE: Helicos sequencing followed with CAGE
P#: Postnatal day #
RL: Rhombic lip
shRNA: short hairpin RNA
TF: Transcription factor
