# Supplementary figures and images for: Identification of novel cerebellar developmental transcriptional regulators with motif activity analysis
Source: BMC Genomics. 2019 Sep 18;20:718. doi: 10.1186/s12864-019-6063-9 (PMC6751898; doi:10.1186/s12864-019-6063-9)

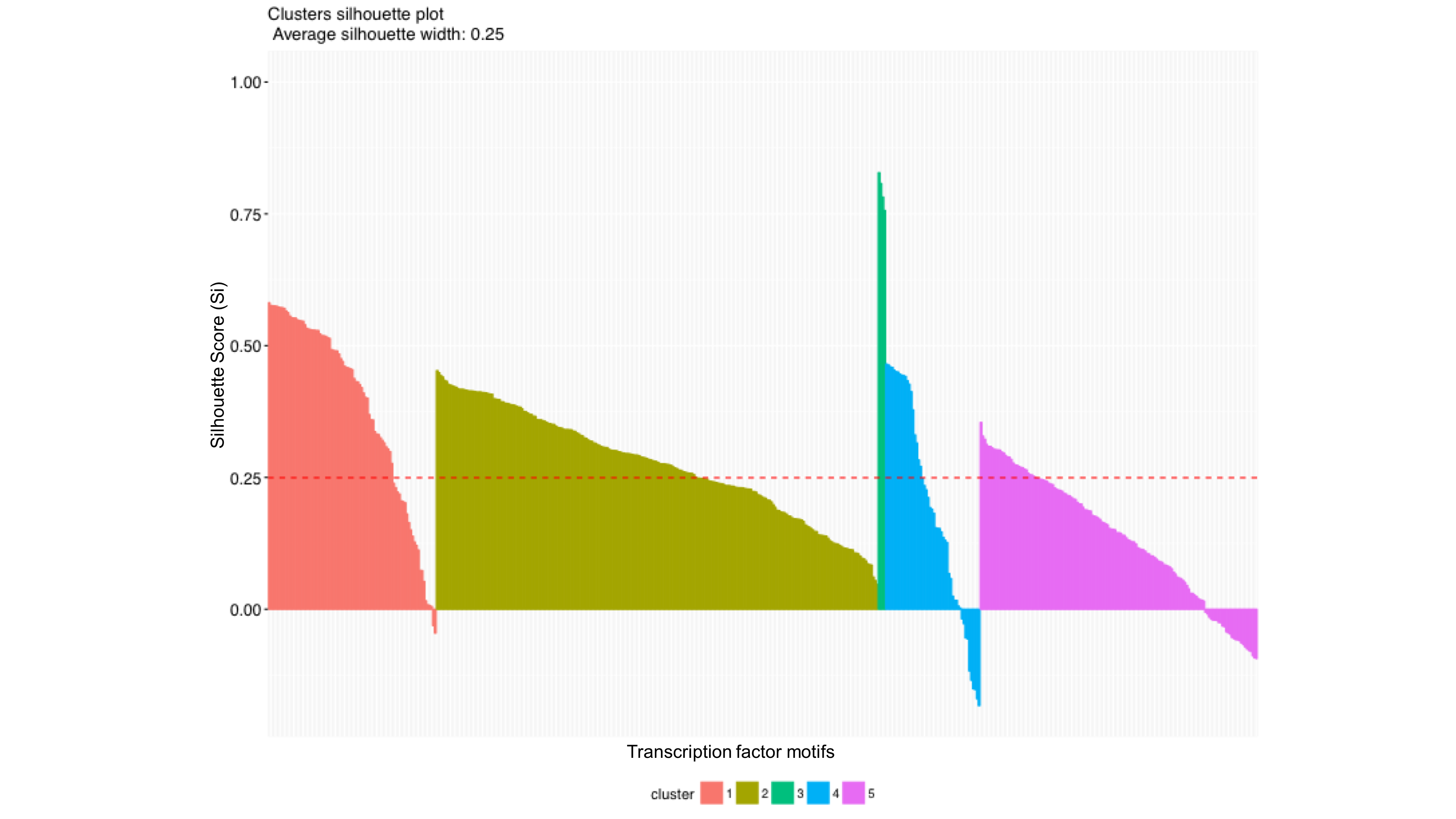

Supplement: Supplementary file 1 — Figure S1. Silhouette plot for k = 5. Y-axis gives information for silhouette score (Si) and each point on the x-axis is a TF motif. The average silhouette score is represented by the red dotted line and is set at 0.26. (TIF 3670 kb) [file 12864_2019_6063_MOESM1_ESM.tif]

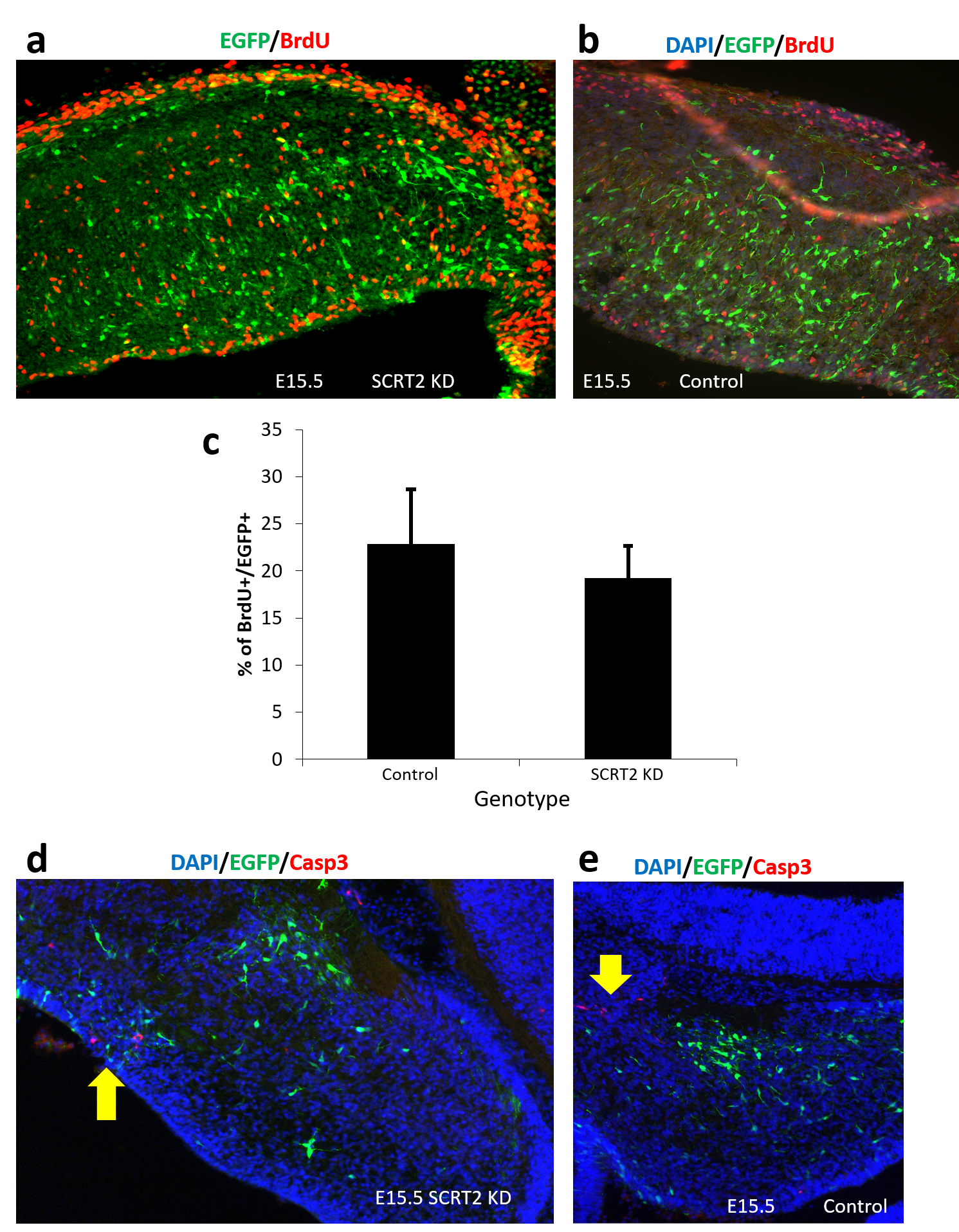

Supplement: Supplementary file 2 — Figure S2. The knockdown (KD) of SCRT2 in the developing cerebellum has no influence on cell proliferation or programmed cell death. Co-label of BrdU (red) and EGFP (green) in E15.5 (a) SCRT2 knockdown and (b) control cerebellum. (c) Quantitative analysis of cell proliferation in control and SCRT2 knockdown cerebella. The percentage of BrdU-positive cells of all EGFP-positive cells between control and SCRT2 knockdown cerebella is insignificant. Co-label of active Caspase-3 (red) and EGFP (green) in E15.5 SCRT2 knockdown (d) and control (e) cerebellum. (TIF 3490 kb) [file 12864_2019_6063_MOESM2_ESM.tif]
