# Supplementary material for: Identification of novel cerebellar developmental transcriptional regulators with motif activity analysis
Source: BMC Genomics. 2019 Sep 18;20:718. doi: 10.1186/s12864-019-6063-9 (PMC6751898; doi:10.1186/s12864-019-6063-9)
Supplement: Supplementary file 3 — Table S1. The predicted targets of the three genes with a knockdown phenotype, from Transfactivity analysis, are shown in the table. In column B is the location of the target and in Column C is the identification of the predicted downstream gene. (DOCX 16 kb) [file 12864_2019_6063_MOESM3_ESM.docx]

| **Gene** | **Genomic position, strand** | **Promoter number @ Gene symbol** |
| --- | --- | --- |
| **Scrt2** | chr14:71042107..71042130,- | p1@Fgf17 |
|  | chr17:63849880..63849947,- | p3@Fbxl17 |
|  | chr3:107889508..107889625,- | p1@Ampd2 |
|  | chr3:122970363..122970418,+ | p1@Sec24d |
|  | chr4:68423102..68423121,- | p@chr4:68423102..68423121,- |
|  | chr5:138052600..138052656,- | p1@Pcolce |
| **Rfx3** | chr11:108286556..108286618,+ | p1@Ccdc46 |
|  | chr11:109512239..109512317,+ | p1@Prkar1a |
|  | chr11:87172513..87172583,- | p1@Ppm1e |
|  | chr12:109661279..109661346,+ | p1@Eml1 |
|  | chr12:109661348..109661367,+ | p2@Eml1 |
|  | chr13:20564559..20564605,+ | p11@Elmo1 |
|  | chr13:43711319..43711360,+ | p1@Rnf182 |
|  | chr13:43711367..43711385,+ | p2@Rnf182 |
|  | chr16:33056542..33056581,+ | p1@Gm11810,p1@Gm14279,p1@LOC100505045,p1@LOC100505110,p1@Rpl35a-ps7,p1@Rpl35a |
|  | chr16:78301918..78302005,+ | p1@Cxadr |
|  | chr17:25965981..25966021,+ | p1@Jmjd8 |
|  | chr17:34973932..34973994,+ | p2@Dom3z |
|  | chr17:36116182..36116253,- | p1@Prr3 |
|  | chr17:57150694..57150749,- | p1@Gtf2f1 |
|  | chr17:57150813..57150828,- | p2@Gtf2f1 |
|  | chr17:85025798..85025852,+ | p1@Dync2li1 |
|  | chr1:174259392..174259438,- | p1@Kcnj9 |
|  | chr1:59294019..59294100,- | p1@Als2 |
|  | chr1:72905308..72905332,- | p@chr1:72905308..72905332,- |
|  | chr2:35516816..35516889,+ | p2@Dab2ip |
|  | chr2:73613398..73613435,- | p2@Chn1 |
|  | chr3:89049081..89049115,- | p2@Trim46 |
|  | chr5:103640270..103640340,- | p1@Mapk10 |
|  | chr5:103854221..103854250,+ | p2@Ptpn13 |
|  | chr5:124928339..124928393,- | p1@Rilpl2 |
|  | chr6:122289815..122289894,- | p1@Phc1 |
|  | chr6:64992569..64992656,+ | p1@Smarcad1 |
|  | chr6:90760112..90760154,- | p5@Iqsec1 |
|  | chr7:17568414..17568499,+ | p@chr7:17568414..17568499,+ |
|  | chr7:77506520..77506570,- | p@chr7:77506520..77506570,- |
|  | chr7:77506609..77506634,- | p@chr7:77506609..77506634,- |
|  | chr7:77506636..77506663,- | p@chr7:77506636..77506663,- |
|  | chr8:112741848..112741904,+ | p1@Ftsjd1 |
|  | chr8:85856315..85856393,- | p1@Elmod2 |
|  | chr9:42280281..42280346,- | p1@Tbcel |
|  | chr9:45179721..45179738,+ | p@chr9:45179721..45179738,+ |
|  | chr9:50425333..50425394,- | p1@AU019823 |
|  | chr9:82723574..82723683,+ | p1@Irak1bp1 |
|  | chrX:133125380..133125405,+ | p2@Tceal3 |
| **Atf4** | chr5:145901234..145901283,+ | p1@Bud31,p1@LOC100045848 |
|  | chr19:43598952..43599070,- | p1@Got1 |
|  | chr8:96910386..96910409,+ | p1@Herpud1 |
|  | chr7:117161815..117161840,+ | p1@Ipo7 |
|  | chr15:76541040..76541095,+ | p1@Lrrc14 |
|  | chr11:120505011..120505076,- | p1@Pycr1 |
|  | chr6:64992569..64992656,+ | p1@Smarcad1 |
|  | chr3:96067248..96067263,- | p2@Hist2h4 |
|  | chr7:117161768..117161781,+ | p2@Ipo7 |
|  | chr6:64992801..64992894,+ | p2@Smarcad1 |
|  | chr9:45793448..45793498,- | p3@Pafah1b2 |
